# Using surrogate biomarkers to improve measurement error models in nutritional epidemiology

**DOI:** 10.1002/sim.5803

**Published:** 2013-04-02

**Authors:** Ruth H Keogh, Ian R White, Sheila A Rodwell

**Affiliations:** 1 MRC Biostatistics UnitCambridge, U.K.; 2 MRC Centre for Nutritional Epidemiology in Cancer Prevention and Survival, Department of Public Health and Primary Care, University of CambridgeCambridge, U.K.; 3 Department of Medical Statistics, London School of Hygiene and Tropical MedicineLondon, U.K.

**Keywords:** biomarkers, measurement error, nutritional epidemiology, regression calibration, structural equation models

## Abstract

Nutritional epidemiology relies largely on self-reported measures of dietary intake, errors in which give biased estimated diet–disease associations. Self-reported measurements come from questionnaires and food records. Unbiased biomarkers are scarce; however, surrogate biomarkers, which are correlated with intake but not unbiased, can also be useful. It is important to quantify and correct for the effects of measurement error on diet–disease associations. Challenges arise because there is no gold standard, and errors in self-reported measurements are correlated with true intake and each other. We describe an extended model for error in questionnaire, food record, and surrogate biomarker measurements. The focus is on estimating the degree of bias in estimated diet–disease associations due to measurement error. In particular, we propose using sensitivity analyses to assess the impact of changes in values of model parameters which are usually assumed fixed. The methods are motivated by and applied to measures of fruit and vegetable intake from questionnaires, 7-day diet diaries, and surrogate biomarker (plasma vitamin C) from over 25000 participants in the Norfolk cohort of the European Prospective Investigation into Cancer and Nutrition. Our results show that the estimated effects of error in self-reported measurements are highly sensitive to model assumptions, resulting in anything from a large attenuation to a small amplification in the diet–disease association. Commonly made assumptions could result in a large overcorrection for the effects of measurement error. Increased understanding of relationships between potential surrogate biomarkers and true dietary intake is essential for obtaining good estimates of the effects of measurement error in self-reported measurements on observed diet–disease associations. Copyright © 2013 John Wiley & Sons, Ltd.

## 1. Introduction

The exposure of interest in nutritional epidemiology is typically the long-term average or ‘usual’ daily intake of a given nutrient, food, or food group. However, there is no gold standard measurement, and studies of the association between dietary intake and disease rely heavily on self-reported measures of intake, which may be subject not only to random errors but also to errors that depend on the true exposure level and on person-specific biases. Errors in measures of dietary intake result in biased estimates of diet–disease associations. Random error causes associations to be underestimated and possible failure to detect associations 1. Systematic error, on the other hand, can result in underestimated or overestimated associations 1. It is important to try to quantify and correct for the effects of measurement error on observed diet–disease associations.

Self-reported measurements of dietary intake are obtained using food frequency questionnaires (FFQs) or using records of actual intake over a day or series of days 2. Types of food record include 24-h recalls, diet diaries, and weighed food records. FFQs are structured questionnaires designed to measure habitual intake of the foods listed by asking individuals to choose their usual frequency of intake from a number of categories offered. FFQs provide a relatively inexpensive method of measuring dietary intake compared with food records, which are time-consuming to process, may require lengthy interviews, and can be burdensome for participants. Hence, the FFQ has typically been used as the main dietary instrument in large prospective studies, whereas food records may be obtained only for a subset of the cohort. Examples include the European Prospective Investigation into Cancer and Nutrition (EPIC) 3 and the National Institutes of Health–American Association of Retired Persons Diet and Health Study 4. FFQ measurements are subject to systematic error, due to the omission of some foods from the questionnaire, the lack of detailed information on portion size, difficulty of accurate recall, and individual tendencies towards biased reporting. Food records are generally considered to provide less biased measures of intake because they measure actual food intake and do not rely on long-term recall, although they are subject to day-to-day variability. More recently, diet diaries have been used to provide the main measure of dietary intake in case–control studies in the UK Dietary Cohort Consortium 5. Measurements from both FFQs and food records are subject to error at the data-processing stage, for example, due to limitations of food databases.

### 1.1. Correcting for error in dietary measurements using regression calibration

Let *T*_*i*_ and *X*_*i*_ denote the true dietary exposure and the observed measurement, respectively, for individual *i*. We assume these to be continuous measurements. We suppose that the diet–disease association is linear, for example, on the logistic scale, and let *θ*_1_ denote the log odds ratio or log hazard ratio as appropriate, which could be estimated directly if *T*_*i*_ could be measured exactly. The observed association found by replacing *T*_*i*_ with *X*_*i*_, denoted 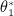, is biased if *X*_*i*_ is subject to measurement errors. For a linear diet–disease association on the appropriate scale (e.g. logistic), we can estimate *θ*_1_ by replacing *T*_*i*_ with *E*(*T*_*i*_ | *X*_*i*_) 1, 6. Under a linear regression model, this is exact, and it has been found to hold approximately under logistic models and proportional hazard models 6, 7. We refer to this method as regression calibration. In many cases, we find a good approximation to the expectation *E*(*T*_*i*_ | *X*_*i*_) by fitting a linear regression model:


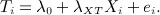
(1)

Sometimes, *X*_*i*_ and *T*_*i*_ are appropriately transformed prior to using this approach.

The estimate of *θ*_1_ found by using *E*(*T*_*i*_ | *X*_*i*_) in place of *T*_*i*_ in the diet–disease model is equal to 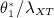 (approximately in the case of logistic and proportional hazard regression). We refer to the correction factor *λ*_*XT*_ as the regression dilution ratio (RDR) and estimate it by 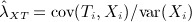.

When *T*_*i*_ cannot be observed even in a validation study, we can still estimate the RDR if additional exposure measurements are available. This is carried out by replacing *T*_*i*_ in (1) with an error-prone but unbiased ‘reference’ measurement, that is, a measurement that is subject only to random error, which may be available in a validation sample within a cohort. This requires a model for the error in the main and reference measurements and a number of assumptions.

Suppose that the error in the main measurement *X*_*i*_ can be modelled as


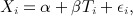
(2)

where the errors *ε*_*i*_ have mean 0, have constant variance, and are independent of *T*_*i*_ and of each other. Parameter *β* represents errors dependent on true intake. When *α* = 0 and *β* = 1, (2) is the classical measurement error model, and *X*_*i*_ is an unbiased measure of *T*_*i*_. Suppose that a second measurement 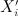 is available, which is subject only to classical measurement error, that is, 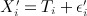. In the case of classical measurement error in the main exposure, 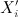 may be a repeat measurement of *X*_*i*_ obtained using the same instrument. Alternatively, if *X*_*i*_ is subject to a nonclassical error, 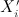 may be a different type of measurement, for example, a less error-prone but more expensive measurement, which provides an unbiased measure of *T*_*i*_ and is available in a validation study. Under the crucial assumption that the errors in *X*_*i*_ and 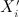 are independent, we can estimate the RDR *λ*_*XT*_ by a regression of 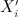 on *X*_*i*_ 1, 8.

We now put this into the context of a nutritional epidemiological study. Most commonly, researchers have compared FFQ measurements (*X*_*i*_) with measurements obtained from food records 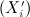 available in a subset of the cohort to correct for the effects of error in the questionnaire measurements using the aforementioned method of regression calibration 9–17. However, there is reason to believe that the assumptions on which this depends are not met, specifically that food record measurements are subject to errors that depend on *T*_*i*_, that is, nonclassical measurement errors, and that errors in FFQ and food record measurements are correlated both with each other and across repeated measurements of the same type.

### 1.2. Using biological measurements

Recovery biomarkers are absolute measures of nutrient intake over a short period (e.g. 24 h) and provide unbiased measures of long-term intake 18. However, these exist only for energy 19, protein, potassium, and sodium 20, 21. One way of using recovery biomarker measurements is to use them as a reference measurement 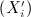 in regression calibration, where the main measurement (*X*_*i*_) is either an FFQ or a food record, under the reasonable assumption that errors in recovery biomarker measurements are purely random and therefore independent of errors in self-reported measurements. Another way to use recovery biomarkers is in a measurement error model defined for FFQ, food record, and recovery biomarker 11, 22–27. This enables estimation of RDRs if an FFQ or food record is used as the main measurement and also investigation of the structure of measurement error in the self-reported measurements, in particular if the self-reported measurements are subject to systematic error depending on *T*_*i*_ (*β* ≠ 1 in (2)) and if errors in the two types of measurement are correlated. Studies using recovery biomarkers have provided evidence that both FFQ and food record measurements are subject to systematic error depending on *T*_*i*_ and the errors in the two types of measurement are correlated. Unfortunately, the scarcity of recovery biomarkers makes such investigations impossible for most nutrients and for all foods.

Some studies have available other biological measurements, referred to as concentration biomarkers, which are correlated with, but not unbiased for, intake of certain nutrients, because they are influenced by other factors such as absorption, metabolism, and individual characteristics 18. In this paper, we use the more general term ‘surrogate biomarker’ to refer to any biomarker that is correlated with the dietary exposure of interest, be that a food or nutrient, and also affected by other factors. Surrogate biomarkers may be obtained relatively cheaply, for example, in stored blood or urine samples. Examples include plasma vitamin E 2, urine or serum phytoestrogens 28, and plasma vitamin C 29, which is used in our later illustration. For the usual case in which there is no recovery biomarker, we can use surrogate biomarkers in measurement error models with FFQ and food record measurements, which allows more flexibility of assumptions made about error in the self-reported measurements 13, 14, 25, 30–34. However, previously proposed models involving surrogate biomarkers still require assumptions that, as discussed later, may be unreasonable.

### 1.3. Plan of the paper

In this paper, we describe a measurement error model for self-reported measurements using surrogate biomarkers. We propose the use of sensitivity analyses to investigate the effects of certain commonly made assumptions about the types of errors in dietary measurements. In Section 2, we give an overview of measurement error models for two and three dietary measurements and describe our extended model for two types of self-reported measurement and a surrogate biomarker. This work was motivated by data on self-reported measures of fruit and vegetable intake from questionnaires and 7-day diaries and a surrogate biomarker (plasma vitamin C) in the EPIC-Norfolk study, a prospective UK study of diet and cancer with over 25000 participants. This study is unique in that diet diary and plasma vitamin C measurements are available for a large number of participants at two time points. Previous studies of measurement error involving food record measurement and biomarkers have been limited by small numbers of subjects. Fruits and vegetables are of major interest in nutritional epidemiological research. In Section 3, we illustrate the proposed model and sensitivity analyses using the EPIC-Norfolk data. To our knowledge, this is the first time that surrogate biomarkers have been considered for use in measurement error models for food intakes rather than nutrient intakes. This is important because the possibility of a recovery biomarker for a food seems remote. The main focus is on estimation of RDRs for use in correcting observed diet–disease associations for the effects of measurement error in the dietary assessment. In particular, in this paper, we show the effects on RDR estimates of deviations from two commonly made assumptions in measurement error models involving surrogate biomarkers. It is also of interest to make comparisons of the degrees of error in FFQ and 7-day diary (7DD) measurements of fruit and vegetable intake. We conclude with a discussion in Section 4.

## 2. Measurement error models

### 2.1. Overview of models for FFQ, food record, and biomarker

Throughout the paper, the following notation will be used: *T*_*i*_ as the true long-term average intake for individual *i*, *Q*_*ij*_ the FFQ measurement for individual *i* at time point *j*, *R*_*ij*_ the food record measurement for individual *i* at time point *j*, and *M*_*ij*_ the biomarker measurement for individual *i* at time point *j*. To simplify some of the notation, we let *X* refer to any of the error-prone measurements, {*Q*,*R*,*M*}.

First, consider a measurement error model for FFQ and food record only, in which the food record is treated as the reference measure for the FFQ, that is, the food record is assumed to provide an unbiased measure of true intake *T*_*i*_:


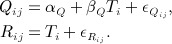
(3)

A number of authors have considered models of a similar form to (3) 9, 13, 15, 17, 25. The assumptions required to identify the parameters of model (3) depend on how many repeated measures of each type are available. If only one measurement of each type is available (*Q*_*i*1_,*R*_*i*1_), we require the assumption that the errors 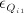 and 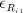 are uncorrelated (or have some known correlation) to estimate the RDR. In this case, the RDR is *λ*_*QT*_ = cov(*R*_*i*1_,*Q*_*i*1_) / var(*Q*_*i*1_), and we can estimate it from a linear regression of *R*_*i*1_ on *Q*_*i*1_. Note, however, that not all of the individual parameters of the model can be estimated in this case, that is, the model is not fully identified. The availability of a second FFQ measurement, so that we have (*Q*_*i*1_,*Q*_*i*2_,*R*_*i*1_), allows us to estimate all of the model parameters only under the additional assumption that the errors in repeated FFQ measurements 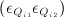 are uncorrelated. This is a highly undesirable assumption. A slight modification is that we can allow a nonzero correlation between the errors in FFQ and the food record measurements made at the same time point only 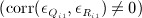. In this case, we do not estimate the RDR *λ*_*QT*_ as described earlier. The addition of a second food record measurement, so that the data are (*Q*_*i*1_,*R*_*i*1_,*R*_*i*2_,*R*_*i*2_), allows estimation of a nonzero correlation between errors in repeated FFQs 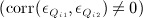, provided we assume that errors in repeated food records are uncorrelated 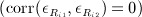 and that errors in FFQ and food record have zero correlation 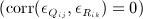. Again, as a slight modification, we can allow for a nonzero correlation between errors in FFQ and food record measurements made at the same time point. Provided that two food record measurements are available, we fully identify the model without a second FFQ measurement under the same assumptions required for the situation with two measurements of each type. If only a single FFQ is available alongside two food records, then of course, we cannot estimate a correlation between errors in repeated FFQs. We can replace all assumptions of zero correlation described here by assumptions that the given correlation takes a known nonzero value. Kipnis *et al*. 15 discussed the use of sensitivity analyses to assess the effects of error correlations on RDRs.

Authors have extended model (3) using recovery biomarker measurements 22–27, 37, and we can write it in the following form:


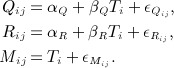
(4)

Under this model, we assume the errors in the biomarker (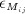) to be independent of those in the self-reported measurements (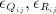), which is reasonable because of the nature of a recovery biomarker. With this assumption, the RDRs for using an FFQ or food record as the main measurement in a diet–disease model are *λ*_*QT*_ = cov(*M*_*ij*_,*Q*_*ij*_) / var(*Q*_*ij*_) and *λ*_*RT*_ = cov(*M*_*ij*_,*R*_*ij*_) / var(*R*_*ij*_), which can be estimated by regressions *M*_*ij*_ on *Q*_*ij*_ and *M*_*ij*_ on *R*_*ij*_, respectively. We can estimate these RDRs using only one measurement of each type. To estimate all of the parameters of model (4) requires a repeated biomarker measurement and the assumption that errors in repeated biomarker measurements are uncorrelated (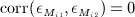), which again is assumed reasonable because of the nature of a recovery biomarker. The preceding assumptions allow estimation of correlation between the errors in FFQ and food record (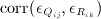), as well as between repeated measures using the self-reported instruments (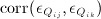, 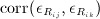), if repeated measures are observed.

The nature of surrogate biomarkers means that surrogate biomarker measurements cannot be assumed to have errors independent of *T*_*i*_; hence, several investigators 13, 14, 25, 31–34, 37 have described measurement error models for FFQ, food record, and a surrogate biomarker of a form similar to


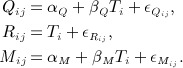
(5)

For identifiability of models of this form, we must have one type of measurement that is assumed to have errors independent of *T*_*i*_, or more generally, systematic errors of a known form. This has invariably been chosen to be the food record measurement despite evidence to the contrary 22, 27. Model (5) is identified under the same assumptions about error correlations as described for model (4). Under model (5), estimation of RDRs is more complex, and we will outline it further below.

In models of the forms described in (3), (4), and (5), some authors have parametrized the model slightly differently by separating the error terms (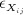) into a random part and a person-specific part 15, 23, 24, 26, 27, 33, 34, 37, at least in the self-reported measurements. For example, we could alternatively write model (5) in the form


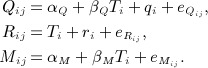
(6)

Spiegelman *et al*. 33 discussed this model in detail. The terms *q*_*i*_ and *r*_*i*_ represent person-specific error in the FFQ and food record, respectively, and 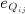 and 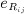 are random errors. Notice that there is no person-specific error term in the part of the model for the surrogate biomarker. When there are at least two measurements of each type (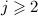), this model is identified under the assumptions that 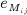 is independent of 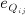 and 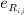 for all *j* and of *q*_*i*_ and *r*_*i*_. The person-specific errors in FFQ and food record may be correlated ( corr(*q*_*i*_,*r*_*i*_) ≠ 0) but are independent of all other error terms. The random error terms in FFQ and food record may be correlated for measurements made at the same time point (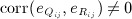) but must be uncorrelated otherwise (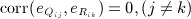).

We can also extend the preceding models to include adjustment for covariates, which is required to estimate RDRs when the underlying exposure–disease model includes covariates (Section 2.4). We further discuss the use of covariates in the following.

For a situation where two or more repeated measurements of each of *Q*, *R*, and *M* are available, Rosner *et al*. 34 proposed extending the surrogate biomarker model in (5) by including time-specific true intake, *T*_*ij*_, denoting average daily intake for individual *i* at time point *j*. Rosner *et al*. 34 separated the errors in measurements into person-specific and random components and also included covariates *Z*_*ij*_. Their model is of the form


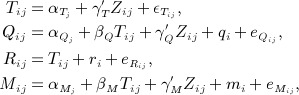
(7)

where *q*_*i*_, *r*_*i*_, and *m*_*i*_ are person-specific error terms and 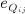, 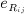, and 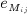 are random error terms. This differs from the previous model in (6) in its inclusion of a person-specific error term for the surrogate biomarker. There are different assumptions regarding error correlations required when a person-specific error term is included for the surrogate biomarker. Model (7) is identified under the assumptions outlined as follows. We assume random error terms 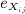 to be independent of *T*_*ij*_ and *Z*_*ij*_, of each other, and of 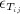. We allow person-specific errors in the self-reported measurements to be correlated ( corr(*q*_*i*_,*r*_*i*_) ≠ 0) but assume them to be independent of *m*_*i*_. Note that the random effect term *m*_*i*_ allows for correlation in the errors between repeated surrogate biomarker measurements. We estimate the variance of *m*_*i*_ at the cost of allowing correlation between random errors in FFQ and food record made at the same time point, that is, it is assumed 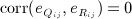. This is in contrast to the model in (6).

Kipnis *et al*. 23, Rosner *et al*. 34, and Spiegelman *et al*. 33 summarized measurement error models that have been used for FFQs and food records using recovery or concentration biomarkers.

### 2.2. The extended model

In this section, we describe a model that extends that of Rosner *et al*. 34. We describe the notation for a study with at least two measurements of each type for each individual. However, provided there are at least two surrogate biomarker measurements, we can calculate all relevant model parameters when FFQ and food record measurements are available at only one time point.

As in the previous discussion, we let *T*_*ij*_ denote the average daily intake for individual *i* at time point *j*, where the expected value of *T*_*ij*_ is a long-term average daily intake, *T*_*i*_. A longitudinal true exposure is appropriate because individual dietary intake clearly fluctuates somewhat over time, and depending on the length of follow-up during which dietary measurements were obtained, it may be desirable to allow for this. For individual *i* at time point *j*, let 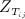 denote a vector of covariates associated with the true dietary intake, and let 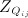, 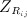, and 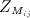 denote vectors of covariates associated with errors in the three types of dietary measurement. The four sets of covariates may differ, and they may be time dependent. The longitudinal measurement error model with covariate adjustment is


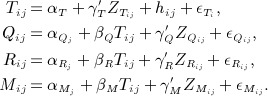
(8)

In the following, we discuss the model further, including assumptions required for identifiability. Table VIII in Appendix A shows how the model parameters are identified. In this paper, we choose not to separate the errors in the dietary measurements into person-specific and random parts, in contrast to some previously suggested models, including models (6) and (7). Appendix A outlines an alternative version of model (8), which includes person-specific error terms.

In (8), the implicit model for long-term average daily intake is 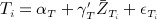, where 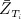 denotes the long-term average covariate value for individual *i*. When the 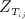 are time constant, the terms *h*_*ij*_ are within-person error terms representing the deviation in true dietary intakes at time point *j* from the long-term average daily intake, *T*_*i*_. In general, the deviation in true intakes at time *j* from the usual intake is 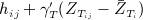. We assume that the *h*_*ij*_ have zero mean, are independent of *T*_*i*_ and of each other ( corr(*h*_*ij*_,*h*_*ik*_) = 0,*j* ≠ *k*), and are independent of all covariates and of errors 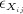. We let 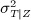 denote the variance of 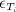 and 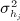 the variance of *h*_*ij*_. We therefore allow the variability in true dietary intakes to differ across time points, through 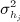. Different types of dietary measurements that are made at the same time point have additional correlation via the *h*_*ij*_ term, compared with dietary measurements made at different time points.

#### 2.2.1. Scaling and intercept parameters

The scaling parameters *β*_*X*_ reflect errors in dietary measurements that are associated with the true intake. We assume these to remain the same over time, although the model allows different intercept terms so that the mean measurements may vary over time. As in the simpler models described earlier, under model (8), we cannot estimate one of the sets of parameters 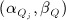, 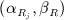, and 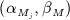, and it has previously been assumed that 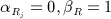. In this paper, we use sensitivity analyses to assess the effects of varying the scaling parameter *β*_*R*_ on estimates of other parameters. We discuss the selection of suitable values for consideration in sensitivity analyses in Section 2.5. When *β*_*R*_ takes a value other than 1, we do not attempt to also fix 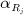 because these parameters are highly dependent on the dietary exposure, with the consequence that 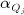, 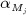, and *α*_*T*_ cannot be estimated. These parameters only affect the other intercept parameters.

#### 2.2.2. Error terms

In model (8), the error terms 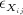 combine both person-specific and random error into one. Our use of combined errors is to some extent a personal preference. However, this formulation for the errors also allows for a little more flexibility in how we define the correlations between errors in longitudinal measurements (Appendix A). This is at the expense, of course, of being able to study person-specific and random sources of error separately. We do not focus on this aspect in this paper. We now outline the features of the error terms in model (8). We assume the errors 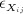 to arise from a normal distribution with mean 0 and variance 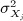. We assume all errors to be uncorrelated with *T*_*ij*_ and with all covariates. We use the following notation for correlations between errors in repeated FFQ and food record measurements given the covariates, where 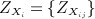:


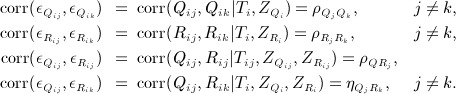
(9)

The error correlation structure summarized in (9) allows the correlation between repeated self-reported measurements made at different time points to change over the course of follow-up and correlations between errors in FFQ and food record to differ according to whether they are made at the same time point or at different time points. There are two prices for estimating the error correlations in (9). These are that we cannot estimate correlations between errors in self-reported and biological measurements or correlation between errors in repeated biomarker measurements. These error correlations therefore need to be handled either by assuming that they are zero or by using sensitivity analyses. We make the assumption that the correlation between errors in self-reported and biological measurements is 0:


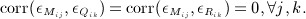
(10)

Inclusion of important covariates in the measurement error model makes this a reasonable assumption. For example, if we are concerned that men and women report dietary intake differently for the same true level of intake and also that sex affects the correlation between true intake and the surrogate biomarker, then conditioning on sex eliminates the concern.

We denote by *ρ*_*MM*_ the correlation between repeated surrogate biomarker error terms:


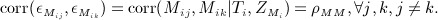
(11)

The inclusion of covariates 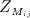 in the extended model may reduce correlation between errors in the repeated surrogate biomarker measurements; however, this will be highly dependent on the surrogate biomarker in question. Many potential surrogate biomarkers are affected by intrinsic individual characteristics, for example, genetics, not easily captured in the covariate adjustment, and it seems unlikely that all sources of correlation between repeated surrogate biomarker measurements could be accounted for solely by true intake and the chosen set of covariates. From the preceding discussion, we are not happy to assume that *ρ*_*MM*_ = 0, and we propose using sensitivity analyses to assess the effects of the value of *ρ*_*MM*_. We discuss the choice of suitable values in Section 2.5.

#### 2.2.3. Covariates

There are three main purposes for covariate adjustment in the measurement error model: to allow individual characteristics to be associated with different degrees of error in self-reported measurements; to make some of the model assumptions more realistic, as discussed in the previous section; and to enable us to use regression calibration when the diet–disease model is adjusted for potential confounders. Relating to the third point, we must include any confounders in the diet–disease model in 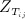. We further discuss this in detail in Section 2.4. It may also be of some secondary interest to investigate the effects of certain covariates on true intake, errors of reporting in the self-reported measurements, and error in the surrogate biomarker.

In model (8), we are unable to estimate the parameters *γ*_*R*_ because of the requirement for identifiability that one of the three measurement types has systematic bias of a known form. We could fix the parameters *γ*_*R*_ in additional sensitivity analyses, but it is difficult to fix a potentially large number of parameters about which we have little information. We can show that the value of *γ*_*R*_ only affects parameters *γ*_*T*_, *γ*_*Q*_, and *γ*_*M*_ (Table VIII). We assume here that reporting in food record measurements *R*_*ij*_ is not dependent on covariates, that is, *γ*_*R*_ = 0. Provided that the variables of concern are included in 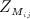, our inability to estimate *γ*_*R*_ does not affect the assumption in (10).

#### 2.2.4. Comparison with earlier models

Finally, we summarize the main differences between our model and that of Rosner *et al*. 34. Our primary extension is the promotion of the use of sensitivity analyses involving the scaling parameter *β*_*R*_ and the biomarker error correlation *ρ*_*MM*_. We allow a random term *h*_*ij*_ in the model for *T*_*ij*_, which allows for true individual intake to fluctuate over time. Inclusion of this term naturally allows for dietary measurements made at the same time point to be more highly correlated than those made at different time points. In our model, the error terms 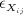 combine both person-specific and random errors and are allowed to have different variances over time. We allow errors in FFQ and food record measurements to have different, presumably higher, correlations when made at the same time point than when made at different time points. By not allowing this, Rosner *et al*. 34 were able to include a person-specific error term in the part of the model for the surrogate biomarker. In model (8), we allow for different sets of covariates to feature in different parts of the model, where Rosner *et al*. 34 assumed just one set of covariates. We should take care in the choice of covariates because the omission of important covariates that are unknown or unmeasured could induce correlations among 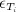, *h*_*ij*_, and 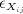 (*j* = 1, …, *J*) and between 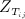 and *h*_*ij*_ or 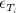. Note that the use of covariates 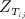 in the measurement error model (8) is not to provide a model for true intake but rather to allow the estimation of covariate-adjusted RDRs and to improve model assumptions.

### 2.3. Fitting the measurement error model

One approach to fitting model (8) is by maximum likelihood assuming a multivariate normal distribution for the dietary measurements conditional on the covariates. The measurements may need to be transformed to meet the assumption of multivariate normality. This method can be computationally intensive if there are many covariates. In the later example, we use the method proposed by Rosner *et al*. 34 in which we first obtain the residuals, 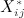, from linear regressions of *X*_*ij*_ on 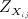 and 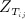 and then use 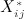 in place of *X*_*ij*_ in model (8) but with the covariate terms omitted. We then estimate parameters *γ*_*T*_ by fitting a mixed-effects linear regression of *R*_*ij*_ on 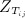 and dividing the resulting estimates by *β*_*R*_. We estimate parameters *γ*_*Q*_ by fitting a mixed-effects linear regression model with response variable *Q*_*ij*_ − *β*_*Q*_*R*_*ij*_ / *β*_*R*_ and explanatory variables 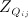. We estimate parameters *γ*_*M*_ in a similar way. To perform these regressions, *β*_*Q*_ and *β*_*M*_ are replaced by their estimated values.

An alternative approach to fitting models of the kind described in the preceding sections is to use an estimating equations approach, which is based on method-of-moments principles, which is described by Spiegelman *et al*. 33. We comment further on the two approaches in Section 3.4.

Usually, only a subset of participants contributing to the diet–disease analysis will have all measurements involved in the measurement error model. Some previous studies have fitted measurement error models using only the data from the subset of individuals with each of the dietary measurements at each time point under consideration, for example, from a validation study, which is usually a small proportion of the total study population 22, 34. Clearly, this is inefficient, and furthermore, it relies on the questionable assumption that measurements are missing completely at random. In this paper, we use a ‘full-cohort’ approach to fitting the measurement error models, including all individuals with an incomplete set of measurements. This likelihood-based analysis is valid and efficient under the weaker assumption that measurements are missing at random 38.

### 2.4. Correction for measurement error in diet–disease models

In this section, we outline how RDRs are estimated under the extended measurement error model. Model (8) allows the dietary exposure to be defined as dietary intake at a particular time point *j*, *T*_*ij*_, or as the long-term average intake, *T*_*i*_. Diet–disease models typically also adjust for potential confounders, say 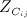, which are assumed to be measured without error. Under the method of regression calibration for a covariate-adjusted diet–disease model, we replace true intake in the diet–disease model by its expectation, conditional on both the observed error-prone measurement and 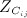. The regression calibration model used to find this expectation may take one of the forms


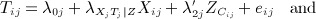
(12)


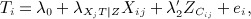
(13)

where *X*_*ij*_ denotes the main error-prone exposure measurement available for all individuals being used to estimate the diet–disease association and 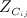 are the covariate measurements from the same time point. Typically, in large-cohort studies, *X*_*ij*_ is an initial FFQ measurement, or in case–control studies, it may be a food record measurement. To reiterate, the RDRs 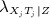 and 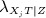 indicate the effect of measurement error in the observed dietary measurement on the estimated diet–disease association, for example, a log odds ratio, when the interest is in time-specific intake or long-term true intake, respectively. The Fibrinogen Studies Collaboration 39 has previously suggested the use of time-dependent measurement error corrections. Model (8) accommodates calculation of the RDRs in the preceding models for FFQ or food record measurements at each time point *j*, provided 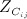 is a subset of 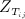. We can show that our inability to estimate the parameters *γ*_*R*_ in model (8) does not affect the RDRs 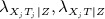.

Estimates of correlations between dietary measurements and true intake are also informative. Note that we can write corr(*Y*_*i*_,*T*_*i*_ | *Z*) = corr(*Y*_*i*_,*X*_*ij*_ | *Z*) / corr(*X*_*ij*_,*T*_*i*_ | *Z*), where *Y*_*i*_ denotes an outcome of interest. The correlations corr(*X*_*ij*_,*T*_*i*_ | *Z*) therefore determine the power of a study to detect diet–outcome associations using *X*_*ij*_. We denote unconditional correlations by 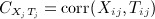 and 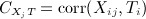 and conditional correlations by 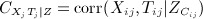 and 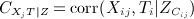.

Appendix B outlines the calculation of the RDRs and correlations for the situation in which 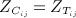.

### 2.5. Sensitivity analyses: choosing values for *β*_*R*_ and *ρ*_*MM*_

We have proposed the use of sensitivity analyses to assess the effects of different values of *β*_*R*_ and *ρ*_*MM*_ on the estimated measurement error model, in particular on the RDRs. In this section, we use results from other studies to inform us about plausible values for these parameters.

Measurement error models for an FFQ, a food record, and a recovery biomarker have been used in a small number of studies to investigate the structure of error in self-reported measures of total energy intake, where the recovery biomarker is obtained using doubly labelled water 19, and intakes of protein, potassium, and sodium, for which urinary measurements provide recovery biomarkers 20, 21. Energy-adjusted protein (‘protein density’) has also been considered. The models used were similar to (4), thus providing estimates of *β*_*R*_. Table I presents the summary of the estimates of *β*_*R*_; they range from 0.34 to 0.81 across studies, nutrients, and type of food record. We could use the results from these studies to inform us about the range of values for *β*_*R*_ that may be plausible in our sensitivity analyses.

**Table I:** Estimates of parameter *β*_*R*_ from measurement error models for the food frequency questionnaire, food record, and recovery biomarker.

Nutrient	Authors	Study	Food record	*β*_*R*_
Protein	Day *et al*. 22	EPIC-Norfolk	7DD	0.81
	Kipnis *et al*. 24	EPIC pilot: France	24HR	0.674
		EPIC pilot: Germany	24HR	0.375
		EPIC pilot: Greece	24HR	0.646
		EPIC pilot: Italy	24HR	0.586
		EPIC pilot: Netherlands	24HR	0.596
		EPIC pilot: Spain	24HR	0.342
		EPIC-Norfolk	7DD	0.614
	Kipnis *et al*. 23 24	MRC pilot: Cambridge	4DWR	0.766
	Kipnis *et al*. 26*	OPEN study	24HR	0.70 (men)
				0.60 (women)
	Schatzkin *et al*. 27*	OPEN study	24HR	0.70 (men)
				0.60 (women)
Energy	Kipnis *et al*. 26*	OPEN study	24HR	0.66 (men)
				0.46 (women)
	Schatzkin *et al*. 27*	OPEN study	24HR	0.63 (men)
				0.42 (women)
Energy-adjusted protein	Kipnis *et al*. 26*	OPEN study	24HR	0.62 (men)
				0.39 (women)
	Schatzkin *et al*. 27*	OPEN study	24HR	0.61 (men)
				0.39 (women)
Potassium	Day *et al*. 22	EPIC-Norfolk	7DD	0.69
Sodium	Day *et al*. 22	EPIC-Norfolk	7DD	0.47

7DD, 7-day diary; 24HR, 24-h recall; 4DWR, 4-day weighed food record; EPIC, European Prospective Investigation into Cancer and Nutrition.

* Kipnis *et al*. (2003) and Schatzkin *et al*. (2003) presented slightly different results from the same study.

We have come across only one study in which estimates of *ρ*_*MM*_ have been obtained. Rosner *et al*. 34 fitted their model (7) to measures of vitamin C intake from repeated FFQ and 7-day diaries, with plasma vitamin C as the surrogate biomarker using data on 323 individuals from the EPIC-Norfolk study. They assumed the 7DD measurement to have no scaling bias (*β*_*R*_ = 1). These authors chose to estimate *ρ*_*MM*_ and instead assumed that correlations between errors in FFQ and 7DD measurements are the same whether or not the measurements were made at the same time point, that is, 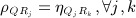. They considered raw-adjusted and calorie-adjusted vitamin C intake, with adjustment for sex, age, body mass index (BMI), height, smoking status, and use of vitamin C supplements in all parts of the model. Without covariate adjustment, the estimates of *ρ*_*MM*_ in models for raw-adjusted and calorie-adjusted vitamin C intake were 0.54 and 0.57, respectively, and in the covariate-adjusted models, the corresponding estimates of *ρ*_*MM*_ were 0.32 and 0.39. Given the evidence from recovery biomarker studies that 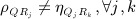, this approach does not seem appropriate in general and, aside from eliciting expert advice for specific surrogate biomarkers, it remains unclear how values of *ρ*_*MM*_ should be chosen for a sensitivity analysis. Using a surrogate biomarker model similar to (5), Wong *et al*. 32 performed sensitivity analyses using values *ρ*_*MM*_ = 0,0.2,0.4 in simulation studies.

## 3. Illustration: plasma vitamin C as a surrogate biomarker for fruit and vegetable intake in EPIC-Norfolk

In this section, we apply the methods outlined in Section 2 to data on intake of fruit and vegetables in the EPIC-Norfolk study, using plasma vitamin C as the surrogate biomarker. EPIC-Norfolk is a cohort of 25639 individuals recruited during 1993–1997 from a population of individuals aged 45–75 years in Norfolk, UK 40. During follow-up, study participants were invited to attend health checks at which dietary intake was assessed using an FFQ and a 7DD and blood samples were provided. Data are currently available from two health checks. Briefly, the first FFQ was mailed to study participants and returned either before or at the date of the first health check, which took place shortly after recruitment. At the first health check, the first day of the diary was completed as a 24-h recall with a trained interviewer and the remainder completed during subsequent days. The second health check took place 3–4 years later, when the FFQ and 7DD were handed out and later returned by post. At each health check, measures of average daily intake of fruit and vegetables (g/day) were derived from the FFQ and 7DD, and plasma vitamin C (mmol/l) was measured within a few days of the blood sample being provided. Bingham *et al*. have described the dietary assessment methods in detail 41.

### 3.1. Use of plasma vitamin C as a surrogate biomarker

Bates *et al*. 29 reported that ‘of all the vitamins, vitamin C exhibits possibly the strongest and most significant correlation between intake and biochemical indices, so that its intake can be predicted with moderate precision from the wide range of biological values that are encountered within the population of a Western country’. Approximately 80–90% of vitamin C intake is absorbed when intake is below 100 mg/day, and absorption saturates at around 140 mg/day 42. Plasma vitamin C has been suggested as a suitable surrogate biomarker for fruit and vegetable intake, as fruits and, to a lesser extent, vegetables are major contributors to dietary vitamin C 43–45, which in turn is correlated with plasma vitamin C 29. However, plasma vitamin C is affected not only by vitamin C intake but also by absorption, metabolism, and genetics 46 and by individual characteristics, including sex, age, smoking status, and BMI 29, 43, 47, 48.

### 3.2. Covariates

In model (8), we allow true fruit and vegetable intake to depend on sex, age, BMI, smoking status, and education level, which is used as an indicator of social class. These were chosen firstly because they are thought to be associated both with true dietary intake and with measurement errors and secondly because in studies of diet–disease associations, they would commonly feature in the set of potential confounders; hence, a regression calibration that is conditional on these variables is of general interest. Intake of fruit and vegetables can also differ across seasons of the year 49. 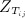 is therefore a vector of covariates for sex, age, BMI, smoking status, education level, and season of measurement.

The vector 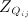 contains covariates for sex, age, BMI, smoking status, and education level, which have been associated with reporting of fruit and vegetable intake on FFQs 50–52. Errors in 7DD measurements could depend on similar covariates 53, 54, but the parameters *γ*_*R*_ cannot be estimated.

As noted earlier, plasma vitamin C has been observed to be affected by sex, age, smoking status, and BMI. In our data, there was a significant upward shift in the plasma vitamin C measurements during the period over which the second health check took place (Table II), which may relate to changes in the laboratory over time; the cause is not clear, and we do not elaborate on this here, but we include an adjustment for month and year of measurement. In model (8), 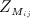 therefore denotes a vector of covariates for sex, age, BMI, smoking status, and month and year of measurement.

**Table II:** Summary of FFQ and 7DD measurements of fruit and vegetable intake and plasma vitamin C measurements in EPIC-Norfolk: number of individuals with each measurement (*N*) and the mean and standard deviation (SD) of the measurements.

Measurement	Health check 1		Health check 2	
	
	*N*	Mean (SD)	*N*	Mean (SD)
FFQ (g/day)	24957	454.8 (258.7)	11732	478.7 (249.6)
FFQ (log-scale g/day)*	24948	5.98 (0.56)	11729	6.04 (0.53)
7DD (g/day)	17293	255.8 (164.3)	2949	296.3 (164.3)
7DD (log-scale g/day)*	17059	5.34 (0.72)	2943	5.52 (0.63)
Plasma vitamin C (mmol/l)	22113	53.0 (19.5)	13373	62.5 (21.1)

FFQ, food frequency questionnaire; 7DD, 7-day diary; EPIC, European Prospective Investigation into Cancer and Nutrition.

* A small number of FFQ and 7DD measurements of zero fruit and vegetable intake are treated as missing when log-scale measurements are used.

The provision to allow different sets of covariates to be associated with true intake, self-reported intake, and errors in the surrogate biomarker was motivated by wanting to allow true dietary intake to be seasonal, whereas it did not seem plausible that season would affect dietary measurement errors. Similarly, we wanted to allow true intake and FFQ reporting, but not errors in the surrogate biomarker, to be associated with education level. Age, BMI, smoking status, and season were recorded at both health checks.

### 3.3. Application of model (8)

The use of a longitudinal exposure *T*_*ij*_ in model (8) was motivated by the long period (3–4 years) between the repeated dietary measurements in the EPIC-Norfolk study. The use of error correlations between dietary measurements, which may differ over time, was motivated by knowledge about the timing and ordering of the self-reported measurements and the thought that self-reporting errors may change over a long period.

We assume a multivariate normal distribution for the dietary measurements to fit model (8). Plasma vitamin C measurements are approximately normally distributed on the untransformed scale, whereas FFQ and 7DD measurements are approximately normally distributed on the log scale. For individual *i* at health check *j* (*j* = 1,2), we let *Q*_*ij*_ and *R*_*ij*_ denote log-transformed FFQ and 7DD measurements, respectively, and *M*_*ij*_ denote plasma vitamin C. Normality also holds approximately for the residuals after adjustment of *Q*_*ij*_, *R*_*ij*_, and *M*_*ij*_ for covariates 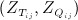, 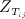, and 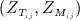, respectively. Estimated RDRs for FFQ and 7DD measurements apply to log-scale fruit and vegetable intake. If untransformed intake is of interest in the diet–disease model, then we can apply a ‘back-transformation’, which is outlined in Appendix B.

A total of 25604 individuals have at least one of six measurements (FFQ, 7DD, or plasma vitamin C at the first or second health check), and all six measurements are available for 2000 individuals. Processing of diet diaries is extremely expensive and time-consuming and is ongoing. The analyses are based on 25275 individuals with at least one dietary measurement and complete covariate information at times of dietary measurement. Table II summarizes the number of individuals with each of the six measurements, and the means and standard deviations of the measurements. We treat very high (75th percentile plus two times the interquartile range) plasma vitamin C measurements (105 at the first health check and 106 at the second health check) as missing because these could be due to the use of vitamin C supplements 55, but quantitative data on supplement use were not available. The covariates, excluding month and year of plasma vitamin C measurements and season of measurement, are summarized in Table III.

**Table III:** Summary of covariates at health checks 1 and 2.

Covariate	Health check 1	Health check 2
Age in years, mean (SD)	58.7 (9.3)	62.3 (9.2)
Body mass index, mean (SD)	26.4 (3.9)	26.7 (3.9)
Sex, *N* (%)		
Male	11455 (45.3)	—
Female	13820 (54.7)	—
Smoking status, *N* (%)		
Never	11608 (45.93)	8272 (48.48)
Former	10700 (42.33)	7325 (42.93)
Current	2967 (11.74)	1467 (8.60)
Education level, *N* (%)		
No qualifications	9285 (36.74)	—
GCSE or equivalent	2596 (10.27)	—
A level or equivalent	10143 (40.13)	—
Degree level or equivalent	3251 (12.86)	—

GCSE, General Certificate of Secondary Education; SD, standard deviation.

For each type of dietary measurement, means and variances differ significantly between health checks 1 and 2 (Table II). In model (8), we therefore allow for different intercepts 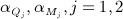 and different error variances 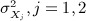.

We performed sensitivity analyses using different fixed values for *β*_*R*_ and *ρ*_*MM*_. As recorded in Table I, Day *et al*. 22 found values of *β*_*R*_ of 0.47, 0.69, and 0.81 for sodium, potassium, and protein, respectively. Sodium intake is thought to be badly measured by the 7DD in EPIC-Norfolk because the instructions provided with the diary were not clear regarding reporting of salt added at the table and during cooking. Higher *β*_*R*_ values for protein and potassium may therefore be more plausible. It was not clear what may be suitable values for *ρ*_*MM*_. We chose *β*_*R*_ = 1,0.75,0.5 and, following Wong *et al*. 32, *ρ*_*MM*_ = 0,0.2,0.4. Although plasma vitamin C is regarded as a promising candidate for use as a surrogate biomarker, as discussed before, it is affected not only by the individual characteristics, which can be accounted for in a set of covariates, but also by intrinsic individual differences.

### 3.4. Results

We show estimates of the derived conditional RDRs and correlations in Table IV, main model parameters in Table V, and parameters associated with covariates in Table VI.

**Table IV:** Model (8): estimated RDRs and correlations between dietary measurements and true intake (standard error) conditional on covariates *Z*_*T*_.

Parameter	*β*_*R*_	*ρ*_*MM*_		

		0	0.2	0.4
RDRs: long-term average intake				
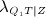	1	0.15 (0.01)	0.19 (0.01)	0.48 (0.01)
	0.75	0.20 (0.01)	0.26 (0.01)	0.64 (0.02)
	0.5	0.31 (0.01)	0.39 (0.02)	0.95 (0.08)
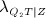	1	0.15 (0.01)	0.20 (0.01)	0.49 (0.01)
	0.75	0.20 (0.01)	0.27 (0.01)	0.66 (0.02)
	0.5	0.32 (0.01)	0.40 (0.02)	0.98 (0.08)
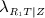	1	0.13 (0.01)	0.17 (0.01)	0.43 (0.01)
	0.75	0.18 (0.01)	0.23 (0.01)	0.57 (0.02)
	0.5	0.27 (0.01)	0.35 (0.02)	0.85 (0.07)
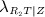	1	0.16 (0.01)	0.21 (0.01)	0.51 (0.01)
	0.75	0.21 (0.01)	0.28 (0.01)	0.68 (0.02)
	0.5	0.32 (0.01)	0.41 (0.02)	1.02 (0.07)
RDRs: time-dependent intake				
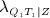	1	0.18 (0.01)	0.24 (0.01)	0.58 (0.02)
	0.75	0.24 (0.01)	0.32 (0.01)	0.78 (0.03)
	0.5	0.36 (0.01)	0.47 (0.02)	1.16 (0.09)
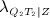	1	0.18 (0.01)	0.23 (0.02)	0.56 (0.05)
	0.75	0.23 (0.02)	0.31 (0.03)	0.75 (0.08)
	0.5	0.35 (0.04)	0.46 (0.05)	1.12 (0.16)
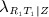	1	0.16 (0.01)	0.21 (0.01)	0.52 (0.02)
	0.75	0.22 (0.01)	0.28 (0.01)	0.70 (0.03)
	0.5	0.32 (0.01)	0.43 (0.02)	1.04 (0.09)
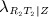	1	0.18 (0.01)	0.24 (0.01)	0.58 (0.02)
	0.75	0.24 (0.01)	0.32 (0.02)	0.77 (0.03)
	0.5	0.36 (0.02)	0.47 (0.03)	1.16 (0.10)
Correlations				
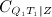	—*	0.34 (0.01)	0.39 (0.01)	0.62 (0.01)
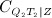	—*	0.34 (0.01)	0.39 (0.01)	0.61 (0.01)
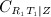	—*	0.40 (0.01)	0.46 (0.01)	0.72 (0.01)
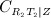	—*	0.43 (0.01)	0.49 (0.01)	0.76 (0.01)
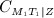	—*	0.79 (0.01)	0.69 (0.01)	0.44 (0.01)
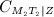	—*	0.71 (0.01)	0.62 (0.01)	0.40 (0.01)

RDR, regression dilution ratio.

* The parameter estimate does not depend on *β*_*R*_.

**Table V:** Model (8): maximum likelihood estimates (standard error) for main model parameters.

Parameter	*β*_*R*_	*ρ*_*MM*_		

		0	0.2	0.4
Scaling bias				
*β*_*Q*_	1	0.66 (0.02)	Same as for *ρ*_*MM*_ = 0	
	0.75	0.49 (0.01)	Same as for *ρ*_*MM*_ = 0	
	0.5	0.33 (0.01)	Same as for *ρ*_*MM*_ = 0	
*β*_*M*_	1	49.96 (1.41)	38.16 (1.34)	15.55 (0.42)
	0.75	37.47 (1.06)	28.63 (1.00)	11.66 (0.44)
	0.5	24.98 (0.70)	19.08 (0.67)	7.78 (0.63)
True intake variance				
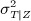	1	0.07 (0.004)	0.09 (0.01)	0.21 (0.01)
	0.75	0.12 (0.01)	0.15 (0.01)	0.37 (0.02)
	0.5	0.26 (0.01)	0.34 (0.02)	0.84 (0.07)
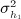	1	0.014 (0.001)	0.019 (0.002)	0.005 (0.01)
	0.75	0.026 (0.003)	0.033 (0.003)	0.082 (0.01)
	0.5	0.057 (0.01)	0.075 (0.01)	0.18 (0.02)
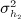	1	0.009 (0.002)	0.012 (0.002)	0.003 (0.01)
	0.75	0.017 (0.003)	0.022 (0.004)	0.053 (0.01)
	0.5	0.037 (0.01)	0.049 (0.01)	0.12 (0.03)
Error variances				
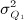	—*	0.25 (0.003)	0.24 (0.003)	0.18 (0.004)
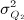	—*	0.25 (0.003)	0.24 (0.004)	0.18 (0.004)
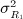	—*	0.41 (0.01)	0.39 (0.01)	0.23 (0.01)
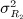	—*	0.34 (0.01)	0.32 (0.01)	0.17 (0.01)
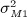	—*	117.06 (4.82)	164.01 (4.97)	253.98 (2.90)
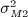	—*	181.40 (5.41)	225.39 (5.34)	309.70 (4.15)
Error correlations				
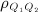	—*	0.65 (0.01)	0.64 (0.01)	0.57 (0.01)
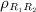	—*	0.56 (0.01)	0.54 (0.02)	0.32 (0.02)
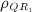	— *	0.45 (0.01)	0.43 (0.01)	0.15 (0.01)
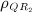	—*	0.51 (0.01)	0.48 (0.01)	0.22 (0.02)
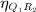	—*	0.38 (0.02)	0.36 (0.02)	0.10 (0.02)
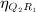	—*	0.44 (0.01)	0.42 (0.01)	0.22 (0.01)

* The parameter estimate does not depend on *β*_*R*_.

**Table VI:** Model (8): maximum likelihood estimates (standard error) for parameters associated with covariates.

Covariate	*γ*_*T*_			*γ*_*Q*_	*γ*_*M*_		
	
	*β*_*R*_ = 1	*β*_*R*_ = 0.75	*β*_*R*_ = 0.5		*ρ*_*MM*_ = 0	*ρ*_*MM*_ = 0.2	*ρ*_*MM*_ = 0.4
Age (years)	0.003 (0.001)	0.004 (0.001)	0.006 (0.001)	0.003 (0.0004)	− 0.23 (0.030)	− 0.21 (0.024)	− 0.18 (0.01)
BMI	− 0.001 (0.001)	− 0.001 (0.002)	− 0.001 (0.002)	0.005 (0.001)	− 0.66 (0.07)	− 0.68 (0.05)	− 0.71 (0.04)
Sex (reference group: males)							
Female	0.17 (0.01)	0.22 (0.01)	0.33 (0.02)	0.12 (0.01)	3.37 (0.53)	5.03 (0.43)	8.22 (0.29)
Smoking status (reference group: never)							
Former	− 0.05 (0.01)	− 0.07 (0.01)	− 0.10 (0.02)	0.02 (0.01)	2.21 (0.55)	1.52 (0.44)	0.18 (0.30)
Current	− 0.39 (0.02)	− 0.52 (0.02)	− 0.78 (0.04)	0.001 (0.01)	8.35 (0.89)	3.60 (0.71)	− 5.49 (0.48)
Education level (reference group: no qualifications)							
GCSE level	0.07 (0.02)	0.10 (0.02)	0.15 (0.04)	− 0.01 (0.01)	—	—	—
A level	0.13 (0.01)	0.18 (0.02)	0.27 (0.02)	− 0.01 (0.01)	—	—	—
Degree level	0.22 (0.02)	0.29 (0.02)	0.44 (0.03)	− 0.03 (0.01)	—	—	—
Season (reference group: spring)							
Summer	0.11 (0.01)	0.15 (0.02)	0.22 (0.03)	—	—	—	—
Autumn	0.07 (0.01)	0.10 (0.02)	0.14 (0.03)	—	—	—	—
Winter	− 0.45 (0.01)	− 0.06 (0.02)	− 0.09 (0.03)	—	—	—	—

BMI, body mass index; GCSE, General Certificate of Secondary Education.

#### 3.4.1. Regression dilution ratios and correlations

Note firstly that our large sample size has enabled us to obtain parameter estimates with high precision. The parameters of the measurement model depend strongly on the assumptions we make about *β*_*R*_ and *ρ*_*MM*_. As a consequence, the degree of correction for measurement error using RDRs is heavily dependent on the model assumptions (Table IV). Depending on what is assumed about both *β*_*R*_ and *ρ*_*MM*_, the RDRs range from 0.15 to 0.98 for FFQ measurements and from 0.13 to 1.02 for 7DD measurements, using the version of the RDR suitable for when long-term intake (*T*_*i*_) is the main exposure in the diet–disease model. RDRs for time-specific exposure (*T*_*ij*_) tend to be a little higher. We might expect this because we would expect FFQ and 7DD measurements to provide better measures of intake at the time of completion than the long-term average intake. Interestingly, the differences between the two types of RDRs are similar for FFQs and 7DDs, suggesting that FFQ reporting is also biased towards recent intake.

For the use of the FFQ as the main measurement, the RDRs are similar at the two health checks, whereas for 7DD, the RDRs are consistently somewhat higher at health check 2. The reasons for this are unclear. Given that the 7DD is generally considered to be a superior measurement to the FFQ and has been found to be in recovery biomarker studies, it is surprising to find that RDRs for 7DD and FFQ are very similar in this study. This suggests that the degree and type of measurement error in FFQ and 7DD may differ considerably across different foods and nutrients.

Correlations of dietary measurements with *T*_*ij*_ are independent of *β*_*R*_. As *ρ*_*MM*_ increases, the correlations for FFQ and 7DD increase, and those for plasma vitamin C decrease. Correlations between FFQ measurements and *T*_*ij*_ are similar at the two health checks, whereas those between 7DD measurements and *T*_*ij*_ are higher at the second health check. 7DD measurements are more highly correlated with true intake compared with the FFQ measurements, indicating higher power to detect diet–disease associations, even though the RDRs are similar.

#### 3.4.2. Main model parameters

The value of *β*_*R*_ affects only the scaling parameters 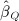 and 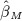 and the variance of true intake, whereas *ρ*_*MM*_ affects all parameter estimates except 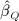 (Table V). The ratio 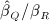 is estimated to be 0.66 regardless of the assumptions.

Estimated error variances for FFQ measurements made at health checks 1 and 2 are practically identical, whereas for 7DD measurements, the variance is higher at health check 1. This could be due to changes in the way individuals reported their dietary intake on the 7DD at the two health checks or to systematic changes in fruit and vegetables intake. Estimated error variances are greater for 7DD than for FFQ, perhaps because of the short-term nature of 7DDs. The estimated variability of errors in plasma vitamin C measurements is markedly higher at health check 2. Recall from Section 3.2 that observed plasma vitamin C measurements were more variable at the second health check.

High correlations between errors in repeated FFQ (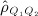) and 7DD (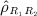) suggest strong individual tendencies to make specific types of reporting error on both instruments. The result that 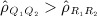 suggests that person-specific errors are more likely to persist across repeated FFQs than across repeated food records. Estimated correlations between errors in the FFQ and 7DD (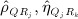) confirm atendency for individuals to make similar types of error on both instruments. Error correlations for FFQ and 7DD at the same health check (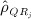) are higher than for measurements made at different health checks (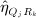).

#### 3.4.3. Covariates

The association of covariates 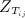 with true intake (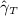) is independent of *ρ*_*MM*_ (Table VI). Increasing age, being female, and a higher level of education are associated with higher fruit and vegetable intake. Former smokers and, to a greater degree, current smokers have significantly lower fruit and vegetable intake compared with never smokers. Intake differs significantly across seasons, being highest in summer and lowest in winter. Estimates 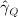 are independent of both *β*_*R*_ and *ρ*_*MM*_, and greater reporting error in the FFQ is significantly associated with increasing age, higher BMI, and being female.

Estimates 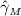 depend on *ρ*_*MM*_ but not *β*_*R*_. Conditional on true fruit and vegetable intake, increasing age, higher BMI, and being male are strongly associated with a lower plasma vitamin C level across all values of *ρ*_*MM*_. When *ρ*_*MM*_ = 0 and 0.2, former and current smokers have higher plasma vitamin C given *T*_*ij*_ compared with never smokers, but when *ρ*_*MM*_ = 0.4, current smokers have a statistically significantly lower plasma vitamin C level than former and never smokers given *T*_*ij*_. Other evidences that smokers have lower plasma vitamin C for the same vitamin C intake compared with nonsmokers 48 lead us to suggest that *ρ*_*MM*_ = 0 and 0.2 may be too low. The situation is complicated, however, because the underlying exposure is fruit and vegetable intake, not vitamin C intake, and also by our inability to estimate parameters *γ*_*R*_, the values of which affect 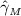. A comparison with analyses without covariate adjustment (results not shown) shows that adjustment for covariates 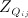 has only a very minor effect on the error variances 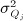 and correlations between errors in FFQ and 7DD. However, adjustment for 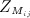 greatly reduced the error variances 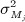, suggesting that the covariates account for a substantial proportion of variability in plasma vitamin C conditional on true fruit and vegetable intake. The former finding suggests that, had we been able to estimate *γ*_*R*_, the results, including estimates 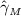, would be only slightly changed.

#### 3.4.4. Which sensitivity values are most plausible?

It is worth considering what might be a plausible set of values for *β*_*R*_ and *ρ*_*MM*_. The earlier results suggest that *ρ*_*MM*_ = 0.4 may be most plausible because it gives the expected negative association between smoking and plasma vitamin C. Higher values of *ρ*_*MM*_ brought significant difficulties in the maximum likelihood estimation, suggesting a poor fit. There is little in these results to favour one value of *β*_*R*_ over another. In the recovery biomarker study of Day *et al*. 22 (Table I), *β*_*R*_ = 0.75 is the mean of the estimated *β*_*R*_ values for potassium and protein intake and may be considered a suitable choice to favour here.

### 3.5. Subset analysis

The use of vitamin C supplements could distort the association between fruit and vegetable intake and plasma vitamin C 55. Although detailed data were not available on supplement use, a binary indicator of vitamin C supplement use has been created from responses to a health and lifestyle questionnaire in which 11382 (45%) were identified as vitamin C supplement users. Plasma vitamin C is significantly higher among vitamin C supplement users: for example, at health check 1, mean plasma vitamin C was 49.0 mmol/l (SD 18.9) among nonusers and 57.8 mmol/l (SD 19.2) among users. We separately refitted model (8) for users and nonusers of vitamin C supplements.

The conditional RDRs from the preceding subset analyses are shown in Table VII for the case where *β*_*R*_ = 0.75 and *ρ*_*MM*_ = 0.4. RDRs are considerably lower for vitamin C supplement users than for nonusers because the estimated variability in true intake and in error correlations is lower among supplement users. Adjustment for vitamin C supplements using a binary indicator, as in 34, is not appropriate because of the large range of doses of vitamin C that individuals may receive from supplements. Future analyses may be able to adjust for vitamin C supplement dose in the measurement error model.

**Table VII:** Subset analyses using model (8): estimated regression dilution ratios (standard error) for the food frequency questionnaire and 7-day diary when *β*_*R*_ = 0.75 and *ρ*_*MM*_ = 0.4.

	Full cohort	Supplement users	Supplement nonusers
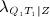	0.78 (0.027)	0.78 (0.077)	0.93 (0.098)
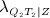	0.75 (0.077)	0.68 (0.168)	0.96 (0.149)
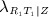	0.70 (0.027)	0.73 (0.076)	0.83 (0.108)
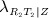	0.77 (0.033)	0.76 (0.085)	0.96 (0.102)

## 4. Discussion

### 4.1. Summary of findings

We have outlined a measurement error model for self-reported dietary intake from FFQs and food records using a surrogate biomarker. We place particular emphasis on using sensitivity analyses to assess the impact of two assumptions that are usually made in measurement error models using surrogate biomarkers: that error in food record measurements is independent of true intake (*β*_*R*_ = 1) and that errors in repeated surrogate biomarker measurements are uncorrelated given true intake and covariates (*ρ*_*MM*_ = 0). The impact of assumptions about *β*_*R*_ in models using surrogate biomarkers does not appear to have been considered previously. Wong *et al*. 32 appeared to have been the only other authors to have considered the effects on the RDR of different values for *ρ*_*MM*_, although they did not give a practical application.

Our extended model was motivated by and illustrated using data from the EPIC-Norfolk study on fruit and vegetable intake, measured using FFQs and 7DD and using plasma vitamin C as the surrogate biomarker. To our knowledge, this is the first use of a surrogate biomarker model for foods rather than nutrients. The model was fitted using data from over 25000 individuals across two time points.

We showed that the choice of values for *β*_*R*_ and *ρ*_*MM*_ can have severe consequences for the estimated RDRs and hence for conclusions about the effects of measurement error on observed diet–disease associations, for example, a log odds ratio. The RDRs for FFQ measurements at health check 1 ranged from 0.15 to 0.95: that is, the measurement error in FFQs could result in anything from a very large attenuation in the log odds ratio to almost no attenuation. Our results indicate that we would make a potentially large overcorrection for the effects of measurement error under the usual assumptions that *β*_*R*_ = 1 and *ρ*_*MM*_ = 0, which resulted in the lowest RDRs. Our results also suggest that for fruit and vegetable intake, the degree of measurement error in FFQs and 7DDs is similar, although the 7DD measurements are a little more highly correlated with true intake.

### 4.2. Using surrogate biomarkers in practice

In light of our results, one might consider what can be gained by using surrogate biomarkers to estimate measurement error models for self-reported dietary measurements. The use of surrogate biomarkers allows us to relax the assumptions that errors in FFQs and food records are independent and that errors in repeated measurements using the same instrument are independent, which in the past have been made in validation studies involving only FFQ and diet diary. However, this comes at the price of assuming that errors in surrogate biomarker measurements are independent of errors in self-reported measurements conditional on covariates, and an inability to estimate the correlation between errors in repeated surrogate biomarker measurement, *ρ*_*MM*_. In the illustration of the model, we showed that different assumptions about the values of these parameters can result in RDRs covering a wide range. This is an important result; it suggests that we should be highly sceptical about results obtained under the usual assumptions and cautious to draw firm conclusions about the degree of error in self-reported measurements unless more information can be obtained about the relationship between the surrogate biomarker in question and true intake. There may, however, be other examples in which the values of the sensitivity parameters do not have such an extreme effect on RDR estimates. For some surrogate biomarkers, we may be happy to assume that *ρ*_*MM*_ is close to 0 given the use of a carefully chosen set of covariates 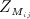.

Experiments to better understand the association between dietary intake and biological measurements will be invaluable in learning more about the values of *β*_*M*_ and *ρ*_*MM*_. Tasevska *et al*. 56 defined a new class of biomarkers called *predictive biomarkers*. These are biomarkers intermediate between recovery and concentration biomarkers. A predictive biomarker has a relationship with true intake that is more complex than that of a recovery biomarker but that is relatively stable and relates to true intake in a dose–response manner 56, 57 such that the relationship can be estimated from a feeding study. There currently appears to exist only one such biomarker, for total sugar intake, which has been used by Tasevska *et al*. 57 to fit models similar to that in (8) (although without some of our extensions) but where the parameters in the part of the model for the biomarker (*M*) were assumed known, having been estimated in a feeding study. In particular, this enables the estimation of intake-dependent error in the food record measurement, *β*_*R*_. Our model in (8) could be applied directly in this situation, but without the requirement for sensitivity analyses. Like recovery biomarkers, predictive biomarkers refer to intake of nutrients, rather than to food groups, such as fruit and vegetables. For surrogate biomarkers for food groups, for example, plasma vitamin C, it may be possible to gain information about the possible range of suitable values to consider for *β*_*M*_ and *ρ*_*MM*_ for use in the measurement error model. Measurement error models such as that described in this paper will become more useful still when more is learnt about potential surrogate biomarkers and when more predictive biomarkers are developed.

### 4.3. Limitations

We outline here some potential limitations of model (8). We assume for identifiability that the terms *h*_*ij*_ are independent across time. This would not be the case if unobserved covariates were associated with the value of *h*_*ij*_. We fitted model (8) again to the EPIC-Norfolk data under the assumption that the correlation between *h*_*i*1_ and *h*_*i*2_ is 0.5, which gave RDRs practically identical to those in Table IV. The *h*_*ij*_ are also assumed independent of the covariates; hence, the model does not allow for the possibility that certain groups of people may have more variability in their dietary intake than others. This assumption may not have been of great importance in the illustration because the estimated within-person variability in true fruit and vegetable intake was small compared with the between-person variability. A related matter is that the variability of errors in dietary measurements is assumed independent of covariates, whereas it seems plausible that error variability may be affected by individual characteristics. Both of these extensions could be incorporated into model (8), and this would result in RDRs being dependent on covariates.

For surrogate biomarkers, it may be the case that the slope *β*_*M*_ in model (8) in fact also depends on individual characteristics and that a random slope may be appropriate, say 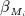. We believe that it would not be possible to estimate such a parameter in this model. However, interactions between covariates 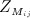 and *T*_*ij*_ in the part of the model for the surrogate biomarker could be incorporated. As before, this would result in RDRs being dependent on covariates.

As outlined in Section 2.3, the measurement error model was fitted by maximum likelihood, assuming a multivariate normal distribution for the dietary measurements. In this example, we used the self-reported dietary measurements on the log-transformed scale. A possible disadvantage of the maximum likelihood approach is that it requires that the dietary exposure is appropriately transformed and then used on the transformed scale in the outcome model of interest (Appendix C). As noted, an alternative approach to fitting the measurement error model is to use the estimating equations approach outlined by Spiegelman *et al*. 33, which is based on the method of moments. The advantages of the estimating equations approach are that no distributional assumptions are required and hence that the choice of scale in the outcome model is not dictated by this. However, the estimating equations approach still makes assumptions about the scale on which the error models are assumed to hold. If the assumptions of multivariate normality are met, then the maximum likelihood approach will offer some gains in efficiency relative to the estimating equations approach. One of our motivations for using the maximum likelihood approach was that under model (8), there are multiple solutions for some parameters under the methods of moments, although Spiegelman *et al*. 33 did suggest a weighted combination of estimates for use in this situation. A comparison of the two approaches under different measurement error settings may be warranted.

Finally, the methods presented in this paper have focused on studies in which a single dietary exposure of interest is measured with error. Analyses in nutritional epidemiology often involve more than one dietary exposure, all of which are likely to be measured with error. In particular, associations are often adjusted for total energy intake. Rosner *et al*. 58 extended the method of regression calibration to enable multivariate measurement error correction. For this situation, multivariate versions of the models such as those considered in this paper are required. These are possible but require assumptions regarding correlations between errors in self-reported measures of different food or nutrients, and models incorporating biomarkers have been scarcely used 59. This presents a major challenge for measurement error correction methods in nutritional epidemiology. See, for example, Thompson *et al*. 17 and Zhang *et al*. 60 for multivariate measurement error models not incorporating biomarkers. Day *et al*. 61 investigated the potential implications of correlated errors and other factors in multivariate measurement error models. In the context of using a recovery biomarker, Carroll *et al*. 62 have considered multivariate measurement error models, although their focus was on using measurements of intake of multiple correlated nutrients to gain precision in a univariate regression calibration.

### 4.4. Conclusions

Estimation of RDRs, and therefore correction of diet–disease associations for the effects of measurement error, is highly sensitive to model assumptions. Depending on the assumptions made, we may conclude that the observed association between fruit and vegetable intake is either grossly underestimated or even somewhat overestimated when using FFQs or food records as the main measurement. The common assumptions that the food record measurement is not subject to scaling bias (*β*_*R*_ = 1) and that errors in repeated surrogate biomarker measurement are independent (*ρ*_*MM*_ = 0) may result in a large overcorrection for measurement error. We should take extreme care when interpreting results from estimated measurement error models and the resulting corrected diet–disease associations. The use of sensitivity analyses seems vital to understanding the potential impact of error in self-reported dietary measurements on observed diet–disease associations. Gaining further knowledge of how potential surrogate biomarkers are associated with true dietary intake and development of more predictive biomarkers are essential for increasing our understanding of how error in dietary measurements affects observed diet–disease associations and in obtaining good estimates of the true effects of diet on health outcomes.

## Appendix A. An alternative version of model (8) including person-specific error terms

We could alter model (8) so that the error terms  are separated into person-specific and random parts. This alternative model takes the form

(A1)

We do not include a person-specific error term for the surrogate biomarker. As already noted with reference to model (7), we cannot allow for both a person-specific error term in the surrogate biomarker and different correlations between FFQ and food record measurements made at the same time point and made at different time points. We assume all error terms in model (14)  to be uncorrelated with *T*_*ij*_ and with all covariates. We assume person-specific errors *q*_*i*_ and *r*_*i*_ to be normally distributed with means 0, variances  and , and correlation corr(*q*_*i*_,*r*_*i*_) = *τ*_*qr*_. We assume that the errors  arise from a normal distribution with mean 0 and variance . The errors  and  (*j* ≠ *k*) have correlation *ρ*_*MM*_, which is to be specified in a sensitivity analysis. As in model (8), we cannot estimate *ρ*_*MM*_ without making undesirable assumptions about other error terms. As before, we also assume *β*_*R*_ to be fixed. Under this model, we must assume that there is no correlation between the random error components in repeated measures of the same type, that is,  and . It follows that we are making an assumption that  and  do not differ depending on the values of *j*,*k*. The formulation of the error under model (8) allows for dependence on *j*,*k* (refer to (9)): this is not important when there are only two repeated measurements but allows for more flexibility if there are more than two repeats. Under model (A1), we use the following notation for correlations between the random error components in FFQ and food record measurements:

(A2)

We cannot estimate both  and . One option is to assume . This assumption allows for an additional correlation between FFQ and food record measurements made at the same time point because we are able to estimate . However, this assumption also has the consequence that we are assuming that the correlation  does not depend on *j*,*k*. The alternative formulation for the error terms used in model (8) allows this bit of extra flexibility in modelling error correlations longitudinally.

**Table AI:** Model (8): observed first and second moments, conditional on covariates, and parameter estimates using method of moments, showing that the model is identified.  and  are the fixed values of sensitivity parameters *β*_*R*_ and *ρ*_*MM*_.

First and second moments	Parameter estimates
	
	*β*_*M*_: solution to
	
	
	
	
	
	
	
	
	
	
	
	
	
	*γ*_*T*_ : regress *R*_*ij*_ on and divide the regression coefficients by
	*γ*_*Q*_ : regress on
	*γ*_*M*_ : regress on

There is more than one set of estimates using methods of moments.

*We cannot estimate the intercepts *α*_*T*_, , and  unless  is specified, and we write these terms as unknown constants here.

## Appendix B. Model (8): regression dilution ratios and correlations

From (8), the conditional RDR for FFQ measurements using exposure *T*_*ij*_ is

We would calculate an unconditional RDR as

where

denotes the variance–covariance matrix for the vectors of covariates  and  denotes the variance–covariance matrix for the vectors of covariates , and  is the matrix of covariances between covariates  and  with (*k*,*l*)th term . The corresponding RDRs using *T*_*i*_, , and  are calculated in the same way as for *T*_*ij*_ but with  omitted in the numerators, and using , the mean of  across time points, in place of . We find the RDRs for the food records by replacing *Q* by *R* and noting that we assume that *γ*_*R*_ = 0.

Conditional and unconditional correlations of FFQ measurements with *T*_*ij*_ are

We calculate  in the same way as  but with  omitted in the numerator and  in the same way as  but with  omitted everywhere except in var(*Q*_*ij*_).

We find the conditional and unconditional correlations of *T*_*i*_ and *T*_*ij*_ with *R*_*ij*_ and *M*_*ij*_ by replacing *Q* by *R* or *M* and noting that *γ*_*R*_ = 0. We approximated variances of RDRs and correlations using the delta method. In the case of the unconditional RDRs and correlations, we assumed that estimates of *γ*_*T*_, *γ*_*Q*_, and *γ*_*M*_ are independent of all other parameter estimates to obtain the variance estimates.

## Appendix C. Measurement error correction on a transformed scale

Suppose that to fit the chosen measurement error model by maximum likelihood, a transformation, say *h*( ⋅ ), is applied to the main dietary measurement *X*. The regression calibration model is therefore of the form

say, where *h*(*X*) is normally distributed. Conditional on *X*, it follows that *h*(*T*) is normally distributed with mean *λ*_0_ + *λ*_*XT*_*h*(*X*) and variance , the variance of the residuals *e*. To perform a regression calibration based on dietary intake on the *untransformed* scale, we need to find the expectation *E*(*T* | *X*) either by integration or by using a Taylor approximation. When *h*(*x*) = log(*x*), the required expectation can be found exactly and is given by

where *λ*_*XT*_ is the RDR found using transformed measurements and *λ*_0_ and  can be estimated using the estimated parameters of the fitted measurement error model. We can therefore estimate the true diet–outcome association by fitting the model

where, for example, for a linear model, *g*(*x*) = *x* and for a logistic model, *g*(*x*) = e^*x*^ / (1 + e^*x*^). It follows that *θ*_1_ can be also estimated by fitting the diet–outcome model using  as the exposure measurement and then dividing the resulting parameter estimate by . This procedure requires estimation of *λ*_0_, which depends on an estimate of *α*_*T*_, which cannot be estimated unless  is specified alongside *β*_*R*_ in the sensitivity analyses. Calculating the variance of the resulting estimate to account for the variability in the estimates of *λ*_0_, *λ*_*XT*_, and  is more difficult than in the case where no back-transformation is required. We could obtain an estimate by bootstrapping.
